# Network Analysis of the Structure of the Core Symptoms and Clinical Correlates in Comorbid Schizophrenia and Gambling Disorder

**DOI:** 10.1007/s11469-022-00983-y

**Published:** 2022-12-27

**Authors:** Roser Granero, Fernando Fernández-Aranda, Zsolt Demetrovics, Milagros Lara-Huallipe, Alex Morón-Fernández, Susana Jiménez-Murcia

**Affiliations:** 1 Department of Psychobiology and Methodology, Universitat Autònoma de Barcelona - UAB, Barcelona, Spain; 2 Ciber Fisiopatología Obesidad y Nutrición (CIBERobn), Instituto Salud Carlos III, Madrid, Spain; 3 Psychoneurobiology of Eating and Addictive Behaviors Group, Neurosciences Programme, Bellvitge Biomedical Research Institute (IDIBELL), L’Hospitalet de Llobregat, Spain; 4 Department of Psychiatry, Hospital Universitari de Bellvitge-IDIBELL and CIBERObn, c/ Feixa Llarga s/n, 08907, L’Hospitalet de Llobregat, Barcelona, Spain; 5 Department of Clinical Sciences, School of Medicine and Health Sciences, Universitat de Barcelona - UB, L’Hospitalet de Llobregat, Spain; 6 Centre of Excellence in Responsible Gaming, University of Gibraltar, Gibraltar, Gibraltar; 7 Institute of Psychology, ELTE Eötvös Loránd University, Budapest, Hungary; 8 Faculty of Psychology, Universitat Autònoma de Barcelona - UAB, Barcelona, Spain

**Keywords:** Network, Schizophrenia, Gambling disorder, Personality, Self-transcendence, Novelty seeking

## Abstract

Few studies have analyzed the clinical profile of treatment-seeking patients with the comorbid presence of schizophrenia (SCZ) and gambling disorder (GD), which warrants new research to assess the network structure of this complex mental condition. The aim of this study was to explore the organization of the symptoms and other clinical correlates of SCZ with GD. Network analysis was applied to a sample of *N* = 179 SCZ patients (age range: 19–70 years, mean=39.5, SD=9.9) who met clinical criteria for gambling disorder-related problems. Variables included in the network were the core GD symptoms according to the DSM-5, psychotic and paranoid ideation levels, global psychological distress, GD severity measures (debts and illegal behavior related with gambling), substances (tobacco, alcohol, and illegal drugs), and personality profile. The nodes with the highest authority in the network (variables of highest relevance) were personality traits and psychological distress. Four empirical modules/clusters were identified, and linkage analysis identified the nodes with the highest closeness (*bridge* nodes) to be novelty seeking and reward dependence (these traits facilitate the transition between the modules). Identification of the variables with the highest centrality/linkage can be particularly useful for developing precise management plans to prevent and treat SCZ with GD.

**Supplementary Information:**

The online version contains supplementary material available at 10.1007/s11469-022-00983-y.

Gambling disorder (GD) is a mental condition defined by persistent, repeated, uncontrollable, and maladaptive patterns of gambling activity, despite the multiple adverse problems and impaired functioning. GD is the only behavioral addiction classified in the latest edition of the Diagnostic and Statistical Manual of Mental Disorders (DSM-5), specifically as a “Substance-Related and Addictive Disorder” (American Psychiatric Association, 2013). The term behavioral addiction is employed for a set of behaviors that individuals become dependent on and crave, in the absence of physical substances. While discussions on whether or not to classify these non-substance addictions as mental disorders continue (Petry et al., 2018), the rationale for grouping GD into the same category than physical addictions into the DSM-5 lies in the growing body of empirical studies showing similarities in many features related to the endophenotype, concretely in the features affecting the neural circuitry of the brains’ reward system (such as lack of control, tolerance, craving, and withdrawal) (Stefanovics & Potenza, 2022). Common treatment responses to interventions planed for addictive conditions have also been observed comparing GD with substance addictions (Hasanović et al., 2021; Li et al., 2020; Loo et al., 2019), as well as comorbidity patterns (Grant & Chamberlain, 2020).

The worldwide prevalence of GD is estimated at around 1% in the latest year of the survey, and between 0.7 and 6.5% of calculated lifetime (Calado & Griffiths, 2016). The large range for the point-estimated prevalences has been related to multiple features, such as the composition of the samples (clinical or population-based), the different assessment tools (screening or diagnostic tools), and the geographical areas (cultural differences and legislation about accessibility to gambling). For example, the lowest rates for problematic gambling have been observed in Oceania (between 0.4 and 0.7%), followed by Europe (between 0.1 and 3.4%), Asia (between 0.5 and 5.8%), and North America (between 2 and 5%) (Calado & Griffiths, 2016). A national multi-center research carried out by a government agency in Spain has estimated the prevalence of GD around 1% in the general population, the percentage of individuals who exhibit moderate- to high-risk behaviors related to gambling around 5%, and around 76% the subjects who reported participation in any gambling activity during the last 12 year (Dirección-General-Ordenación-Juego, 2017). This study also outlined significant gender differences, being the ratio of men versus women treatment seeking for gambling problems around 9:1.

Schizophrenia (SCZ) is a mental disorder that presents significant disability, characterized by a heterogeneous constellation of cognitive and behavioral symptoms associated with high affectation in all areas of life (including personal, family, social, educational, and occupational). According to the DSM-5 taxonomy, typical SCZ symptoms are delusions, hallucinations, disorganized speech, disorganized or catatonic behavior, and other negative mood states (such as diminished emotional expression) (American Psychiatric Association, 2013). Neurological studies differentiate between positive versus negative symptoms (Correll & Schooler, 2020; Marder & Galderisi, 2017; McCutcheon et al., 2019): (a) the positive symptom profile is defined by the disorganization of thinking and behaviors, and is reflected as an excess or distortion of normal functions (such as delusions, hallucinations, paranoia, or disorderly communication); and (b) negative symptoms are related to the motivation/interest and verbal/emotional areas, and are reflected as a diminution of normal behaviors (such as anhedonia, apathy, blunted affect, alogia, or avolition). Updated epidemiological reviews have shown that approximately one in 150 individuals are diagnosed within the psychosis disorder spectrum (SCZ and other forms of non-affective psychosis) at some point of their lifetime (Moreno-Küstner et al., 2018). And according to the latest report published by the World Health Organization, SCZ affects approximately 24 million people worldwide (around 1 in 300, 0.32%), the rate among adults being 1 in 222 (0.45%) (World Health Organization, 2022).

## Dual Presence of GD with SCZ

Epidemiological and clinical studies have observed multiple psychiatric conditions that are comorbid with GD (Dowling et al., 2015; Sundqvist & Rosendahl, 2019; Yakovenko & Hodgins, 2018; Yau & Potenza, 2015), including psychopathologies within the psychotic spectrum (SCZ as well as schizoaffective, schizophreniform, paranoid, and other disorders). It has been observed that the likelihood of problematic gambling behavior is at least 3 times higher among patients with psychosis compared with control population-based samples (Haydock et al., 2015), and gambling-related harm has been reported in the range of 12 to 30% of patients with psychotic disease (Aragay et al., 2012; Desai & Potenza, 2009).

It is also common for people with SCZ to exhibit other comorbid symptoms, including substance-related disorders and/or other behavioral addictions (Johnstone et al., 2022). It has been found that about half of those with SCZ use drugs, usually as coping mechanisms to deal with negative mood states (depression, anxiety, boredom, and loneliness), and during the course and prognosis of SCZ, a global prevalence of around 42% has been found for any substance use disorder (illicit drugs or alcohol) (Hunt et al., 2018). Unfortunately, the presence of comorbid conditions in patients with severe mental illness like SCZ is commonly underdiagnosed and treated (Onyeka et al., 2019), which might lead to interference with antipsychotic medication, less adherence to interventions or even to aggravation/exacerbation of the psychotic symptoms. Treatments of SCZ (typically antidopaminergic antipsychotics, complemented [or not] with psychotherapy) have basically been focused on managing the positive symptoms, proving efficacious for relieving these signs and reducing the risk of hospitalization and relapse (Bighelli et al., 2021; de Bartolomeis et al., 2022; Kishimoto et al., 2021). However, fewer effects have been obtained for negative and cognitive symptoms (such as processing speed, problem-solving, reasoning, and attention) and other psychological problems that are comorbid with SCZ (Haddad & Correll, 2018; Spark et al., 2022). It has also been found that aripiprazole, an antipsychotic (a partial dopamine agonist) used for the treatment of various mental disorders (including SCZ), has been related with the onset of gambling-related problems (Corbeil et al., 2019, 2020). Due to the nature of the dopamine dysfunction found in addictions (substance-related disorders and gambling disorder) (Lachance et al., 2019), pharmacovigilance studies are currently focusing on this association (Grall-Bronnec et al., 2016).

Research in samples characterized by comorbid psychiatric profiles has sought to identify explanatory mechanisms of the dual presence of GD with SCZ. It has been observed that impulsivity (including motor, cognitive, and affective dimensions) can act as a transdiagnostic construct (Hodgins & Holub, 2015; Kräplin et al., 2014; Lee et al., 2013; Ouzir, 2013). Alterations to brain and neurobiological processes have also been proposed as an explanation for the reciprocal relationships between GD and SCZ (mainly disturbances in the motivation-reward and neurotransmitter systems including dopamine, serotonin, or glutamate) (Clark et al., 2019; Howes et al., 2015; Leicht et al., 2020; Potenza & Chambers, 2001; Ruiz et al., 2020; Selvaraj et al., 2014; Zack et al., 2020). Other psychological variables contributing to the dual presence of GD with SCZ are male sex and young age (Gin et al., 2021; Welte et al., 2015), typical cognitive distortions related with the gambling activity (di Trani et al., 2017; Lawlor et al., 2020; Liu et al., 2020; Livet et al., 2020; Mallorquí-Bagué et al., 2018a, b; Yakovenko et al., 2016), severe difficulties in emotion regulation (Szerman et al., 2020), and personality profiles defined by high harm avoidance and low levels of self-directedness (Black et al., 2012, 2013; Sundqvist & Wennberg, 2015).

However, empirical studies focused on the dual pathology of GD with SCZ are scarce and limited. One reason for this is that one of the usual exclusion criteria in GD research is the existence of SCZ, and vice versa (many clinical studies focused on SCZ exclude other severe psychopathological forms such as addictions). Moreover, in clinical practice, and despite the proven reciprocal relationship between GD and SCZ (Cassetta et al., 2018; Corbeil et al., 2019), it is common not to assess the gambling behavior profiles of patients undergoing treatment for SCZ (clinicians usually consider SCZ to be the “primary” diagnosis and prescribe treatments that are specifically focused on monitoring positive and cognitive symptoms) (Fortgang et al., 2018, 2020). And since the presence of GD may not be reflected in the medical histories of individuals seeking treatment for SCZ, an accurate measure of the prevalence of the dual condition of SCZ plus GD remains unknown, as well as the structure of the clinical profile of this complex condition.

In summary, patients who exhibit GD plus SCZ are a highly vulnerable group because of the significant morbidity and disability caused by the complex structure of symptoms. But this high-risk population remains understudied, and new research is required to explore the underlying mechanisms of its clinical profiles. The objectives of this network study were to examine the structure of the core symptoms and other clinical correlates of GD and SCZ, as well as to identify the central nodes, those with the highest closeness and the existence of empirical modules-clusters of symptoms.

To our knowledge, this is the first study to have explored the network basis among patients with the dual presence of GD with SCZ; hence, we were unable to define empirical hypotheses regarding the most relevant nodes or those with the greatest linkage. However, according to an integrative model of mental illness based on the interaction between multi-level mechanisms, which posits that psychopathological states can be conceptualized as interactive systems of mutually reinforcing groups of symptoms that contribute to the onset and progression of diseases (Robinaugh et al., 2020), we expected (a) the existence of distinct modularity classes formed by nodes (symptoms/features) more densely connected together than to the rest of the network and (b) the identification of nodes referred with emotional withdrawal, impulsivity, and difficulty in socialization, as central and bridge nodes.

## Material and Methods

### Participants

The sample included *N*=179 consecutive treatment-seeking patients of the Pathological Gambling Outpatient Unit, Bellvitge University Hospital (Barcelona, Spain). This is a tertiary treatment service specialized in the assessment and treatment of gambling disorder and other behavioral addictions.

Participants were recruited between January 2005 and March 2022 (this long period was required to generate a large enough sample for statistical analyses). Inclusion criteria were age 18+ years, and meeting clinical criteria for GD and SCZ. Exclusion criteria were the presence of an organic mental disorder, intellectual disability, other mental disorder whose symptoms evolve paranoid and psychotic ideation, or neurodegenerative disorder (such as Parkinson’s disease) (the presence of these conditions meant the measurement tools could not be used due the low reliability of the responses).

Most of the 179 participants were men (*n*=163, 91.1%), single (*n*=123, 68.7%), unemployed (*n*=140, 78.2%), reported primary education levels (*n*=116, 64.8%), pertained to a low social position index (*n*=129, 72.1%), and were born in Spain (*n*=168, 93.9%) (Table 1). Age was in the range of 19 to 70 years old (mean age was 39.5 years, SD=9.93). Mean onset of the GD was 26.5 years (SD=9.85), and mean duration of the GD-related problems was 6.8 years (SD=6.86).

**Table 1:** Descriptive of the variables of the study

Sociodemographic		*n*	%	GD measures	Mean	SD	
Sex	Women	16	8.9%	Onset GD (yrs-old)	26.50	9.85	
	Men	163	91.1%	Duration GD (years)	6.85	6.86	
Marital	Single	123	68.7%	DSM-5 criteria:	n	%	
	Married	34	19.0%	A1: Gambling with increasing amounts of money	134	74.9%	
	Divorced	22	12.3%	A2: Restless-irritable when stop gambling	158	88.3%	
Education	Primary	116	64.8%	A3: Repeated efforts to control-stop gambling	166	92.7%	
	Secondary	58	32.4%	A4: Preoccupied with gambling	135	75.4%	
	University	5	2.8%	A5: Often gambles when feeling distressed	128	71.5%	
Employed	Unemployed	140	78.2%	A6: Chasing one’s losses	142	79.3%	
	Employed	39	21.8%	A7: Lies to conceal the extent of gambling	161	89.9%	
Social position	Mean-high	3	1.7%	A8: Has lost relationships, job, education	160	89.4%	
	Mean	7	3.9%	A9: Relies related with financial issues	150	83.8%	
	Mean-low	40	22.3%	Preference	Only non-strategic forms of gambling	136	76.0%
	Low	129	72.1%		Only strategic forms of gambling	9	5.0%
Born in…	Spain	168	93.9%		Mixed forms of gambling	34	19.0%
	Other	11	6.1%	Modality	Only land-based	165	92.2%
Chronological age		Mean	SD		Only online	7	3.9%
Age (yrs-old)		39.53	9.93		Mixed	7	3.9%
Psychopathology (SCL-90R)		Mean	SD	Debts due to GD (yes)		80	44.7%
Paranoid ideation		1.30	0.85	Illegal behavior (yes)		53	29.6%
Psychotic ideation		1.22	0.89			Mean	SD
Global distress (GSI)		1.34	0.74	SOGS total score		10.73	2.74
Personality (TCI-R)		Mean	SD	Substances		n	%
Novelty seeking		108.77	10.19	Tobacco		140	78.2%
Harm avoidance		108.90	13.49	Alcohol		31	17.3%
Reward dependence		94.13	11.43	Illegal drugs		26	14.5%
Persistence		103.09	17.72				
Self-directedness		120.99	17.39				
Cooperativeness		126.18	14.07				
Self-transcendence		64.98	13.80				

Regarding gambling profile, the prevalence of patients achieving each DSM-5 criterion was between 71.5% for symptom-5 “often gambles when feeling distressed” and 92.7% for symptom-3 “repeated unsuccessful efforts to control-stop gambling.” Regarding the preferred gambling activity, 76.0% of patients reported engaging in only non-strategic forms of gambling (this category encompasses gambling that involves little decision-making or skill, and hence no influence on the outcome, such as slot-machines, bingo, and lotteries) (Jiménez-Murcia et al., 2020). The percentage of patients who reported engaged in only strategic forms of gambling (in which gamblers attempt to use their ability to predict the outcome, such as poker, sports/animal betting, and craps) was 5%, and mixed types were reported by 19% of the participants. Regarding the modality of gambling, 92.2% did so in person, 3.9% did so online, and 3.9% reported mixed modalities. The presence of gambling-related debts was reported by 44.7% of patients, and gambling-related illegal behaviors (such as robbery or theft) were observed among 29.6% of patients.

The point prevalence of substance use was 78.2% for tobacco, 17.3% for alcohol, and 14.5% for illegal drugs. For the SCL-90R measures, the number of patients in the clinical group in the paranoid ideation scale was *n*=102 (57.0%), in the psychotic ideation *n*=122 (68.2%), and in the GSI *n*=132 (73.7%).

Table S1 (supplementary material) displays the frequency distribution for the variables of the study among women and men sub-samples. No statistical differences were found comparing women versus men.

### Materials

#### Symptom Checklist-Revised (SCL-90-R) (Derogatis, 1994)

This is a widely used tool that was initially developed to assess the presence and level of a large set of symptoms and other psychological problems. It contains 90 items arranged in nine primary scales (somatization, obsessive-compulsive, interpersonal sensitivity, depression, anxiety, hostility, phobic anxiety, paranoid ideation, and psychotic ideation) and three secondary global indices (global severity index, [GSI], total positive symptoms [PST], and positive discomfort index [PSDI]). The version used in this study (Spanish version) has been proven to have good psychometric indexes (Gonzalez De Rivera et al., 1989). This study used two primary scales for the assessment of the symptoms related to the SCZ condition (paranoid ideation and psychotic ideation), since no other specific measure of the SCZ symptom level and the SCZ functional level were available, and it was not considered adequate to perform a network excluding SCZ indicators. In addition, the global psychology distress (GSI) was used as a measure of the global distress in the sample. The internal consistency in this study for these three scales was from good to excellent: Cronbach-alpha α=0.780 for paranoid ideation, α=0.895 for psychotic ideation, and α=0.982 for GSI.

#### Temperament and Character Inventory-Revised (TCI-R) (Cloninger et al., 1994)

This tool was developed to assess personality profile based on Cloninger’s multidimensional model. The questionnaire includes 240 items and covers 4 dimensions of the individual’s temperament (novelty seeking, harm avoidance, reward dependence, and persistence) and 3 dimensions of the individual’s character (self-directedness, cooperation, and self-transcendence). Novelty seeking is a measure of the exploratory excitability, extraversion impulsivity levels (example of an item: “I do things just for fun”). Harm avoidance measures anticipatory worry, fear of uncertainly, shyness, and fatigability (items like “I worry more than most people that something might go wrong in the future”). Reward dependence measures dependence and openness to warm communication (“I like to please people as much as I can”). Persistence measures eagerness of effort, work hardened, and perfectionism (“I’ve been called a workaholic because of my enthusiasm for working a lot”). Self-directedness measures responsibility, purposeful, self-acceptance, and resourcefulness (“I almost always feel free to choose what I want to do”). Cooperativeness measures social acceptance, empathy, and helpfulness (“I tend to accept others as they are”). And self-transcendence measures self-forgetful and spiritual thinking patterns (“I feel a powerful sense of bonding with all the things around me”). The version used in this study (Spanish adaptation) has been shown to have good psychometric indexes (Gutiérrez-Zotes et al., 2004). The internal consistency in the study sample was between adequate (α=0.705 for novelty seeking) and very good (α=0.867 for persistence).

#### South Oaks Gambling Screen (SOGS) (Lesieur & Blume, 1987)

This tool was designed as a measure to identify the presence of probable, problem, and non-problem gambling, and it has usually been used as a measure of gambling symptom severity. It consists of 20 items aimed at discriminating between probable pathological, problem, and non-problem gambling. The version used in this study (Spanish adaptation) has been shown to have good psychometric indexes (Echeburúa et al., 1994). The internal consistency in this study was adequate, α=0.738.

#### Diagnostic Questionnaire for Pathological Gambling (According to DSM Criteria) (Stinchfield, 2003)

This is a self-report tool formed by 19 items coded in a binary scale (yes-no). It was used to assess the presence of GD according to the DSM-IV-TR (American Psychiatric Association, 2000). This DSM-IV measure has been adapted to measure DSM-5 diagnostic criteria for GD (American Psychiatric Association, 2013) by removing the illegal acts criterion and using the cutoff score of 4 symptoms-criteria. The version used in this study (Spanish adaptation) has been shown to have good psychometric indexes (Jiménez-Murcia et al., 2009). The internal consistency for this scale in the study sample was adequate, α=0.785.

#### Diagnosis of SCZ

All the participants in the study were referred to the treatment unit from local Primary Care Centers and Community Mental Health Centers whose clinicians had identified the presence of GD. The presence of SCZ was diagnosed after assessment by psychiatrist specialists in the treatment of this mental condition, based on DSM-IV and DSM-5 criteria.

#### Semi-structured Clinical Interview

This tool was used to assess additional information, including socio-demographics (sex, marital status, education level, employment status, and social position) and gambling problem-related variables (such as the age of onset and duration of the GD, and the presence of accumulated debts due to gambling behaviors). In this study, the social position index was calculated according to Hollingshead’s Four Factor Index, which provides a classification based on four domains (Hollingshead, 2011): marital status, retired/employed status, educational attainment, and occupational prestige. This semi-structured interview was also used to identify gambling preferences (only non-strategic, only strategic, or mixed), gambling modality (only in person, only online, or mixed), and the use of substances (patients reported the consumption of tobacco, alcohol, and other illegal drugs). This complete tool has been described elsewhere (Jiménez-Murcia et al., 2006).

### Ethics

The study was carried out in accordance with the Declaration of Helsinki of 1975, as revised in 2000. The participants in this study were recruited for different research projects approved by the Ethics Committee of Bellvitge University Hospital (Refs: PR241/11, PR286/14, PR329/19, PR338/17 and PR393/17). All subjects were informed about the research and they all provided informed consent (the acceptance rate was 100% of all the consecutive patients of the treatment unit who met the inclusion criteria).

### Procedure

Data analyzed in this study correspond to a cross-sectional design. In addition to the assessment of the clinical and sociodemographic variables included in the semi-structured interview, the clinicians confirmed the diagnosis of GD provided by the *Diagnostic Questionnaire for Pathological Gambling*, and helped the participants to complete the self-report questionnaires to guarantee that they adequately understood all the items and completed the tools. All the clinicians involved in the recruitment of the sample pertained to the treatment unit, were specialized in the GD area, and had great experience in the assessment and treatment of patients seeking for problematic and disordered gambling.

Throughout the CBT for GD, SCZ patients maintained their antipsychotic medication plans, following the guidelines and management of the clinicians from the Primary Care Centers or Mental Health Centers.

### Network Approach

Network theory offers an outstanding methodological platform to explore complex system of reciprocal interactions (based on the graph theory), as well as to visualize intricate multifaceted phenomena (Borsboom, 2017; Borsboom et al., 2018; Borsboom & Cramer, 2013; Epskamp et al., 2018; Hevey, 20218; McNally, 2016). In the psychiatric area, the network methodology proposes the study of mental disorders as complex phenomena resulting from the underlying interactions between causally connected symptoms/symptoms (which include biological, psychological, and social aspects) (Boschloo et al., 2015; Goekoop & Goekoop, 2014). This approach has proved being useful for discovering the symptoms/features of greatest relevance to the onset and progression of mental problems (“central nodes”) (Fried et al., 2017; Fried & Cramer, 2017). And since this approach does not consider the existence of any single latent entity as the cause of concrete disorders (as is the case with categorical taxonomies), boundaries between diagnostic categories become vague, and comorbid profiles (overlapping symptoms) are explained by means of “transition/bridge” nodes (symptoms that facilitate the paths between structures) (Braun et al., 2018; Cramer et al., 2010).

In the clinical research area, the underlying structure of variables through is displayed through two elements: (a) nodes (symptoms and other sociodemographic-clinical variables), which are represented through circles; and (b) edges (relationships between variables), which are represented as connecting lines (Borgatti et al., 2009). The effect size of the associations between the nodes is visualized in the thickness of the edges (for example, a large effect size is reflected through a thick edge, while two unrelated variables are reflected by two unconnected nodes). The effect size of the associations can be calculated using several statistical procedures, such as the partial correlations matrix, adjusted regression coefficients, adjusted odds ratio coefficients, or factorial loads (these parameters need to be adjusted to avoid biases due to the impact of possible confounding variables) (Bringmann et al., 2013; Clifton & Webster, 2017; Hevey, 20218).

### Statistical Analysis

The network analysis was conducted with Gephi 9.2 for Windows (Bastian et al., 2009) (available at http://gephi.org), a software package that was specifically developed for exploring and visualizing networks within datasets. The system enables a powerful spatialization process and the computation of essential parameters of centrality, density, and modularity clustering.

In this study, a network for the total sample (*n*=179) was obtained. Due the strong asymmetry for the sex distribution, a complementary network among the men sub-sample was also identified (*n*=179). Nodes analyzed in the study were the nine DSM-5 criteria for GD, severity of the GD (total SOGS as a measure of symptom level, the presence of debts, and illegal behavior), psychotic and paranoid ideation levels (according to the SCL-90R), the global psychology distress (SCL-90 GSI), the presence of substance addictions (tobacco, alcohol, and illegal drugs), and personality profile (measured with the TCI-R). The effect size and the signal of the edges were calculated through partial correlations between nodes. The initial data structure for the network resulted in 25 nodes and 300 potential edges, most of which had very low weights (partial correlations around 0). To simplify this initial complex structure, edges that did not reach significance (*p*<0.05) were excluded, resulting in a final structure with 91 edges (around 30.3% of all potential connectors).

The prominence and linkage capacity of the nodes within a network can be measured through distinct indexes, such as centrality and closeness (Epskamp et al., 2018). In this study, node-level relevance within the network was measured with centrality parameters, concretely eigenvector centrality, and authority (these coefficients are obtained from the weighted sum of centrality measures of all nodes connected to a node). High centrality indexes indicate that the information contained in a concrete node is highly valuable for the whole graph.

The node-level linkage was measured in the study using the closeness parameters (which measure how close the node is to all the other nodes in the graph). High closeness indexes indicate a short average distance between one node and all the other nodes, and therefore, nodes with high closeness values have a high capacity to promote relevant changes in other parts of the network (and they are also highly vulnerable to the impact of modifications to any part of the structure).

The presence of empirical clusters of nodes (also called communities or modules in Gephi) was automatically identified (Blondel et al., 2008), as well as the clustering coefficient for each node (these values measure the importance of a node within its community). Node clusters are identified for those variables that are most highly interconnected to each other and poorly connected with nodes outside the cluster.

Other graph distance measures used in the study were (a) the (average) path length, calculated as the mean of the shortest paths between all pairs of nodes (this value represents a measure of the efficiency of information transport in the network), and (b) the diameter, calculated as the greatest distance between the two furthest nodes (representing the maximum eccentricity of any vertex in the graph) (Brandes, 2001). The density of the graph was also estimated as the number of connections divided by the number of possible connections, which provides a measure of how close the network is to being complete (a complete graph includes all possible edges and achieves a density measure equal to 1).

## Results

**Fig. 1:**
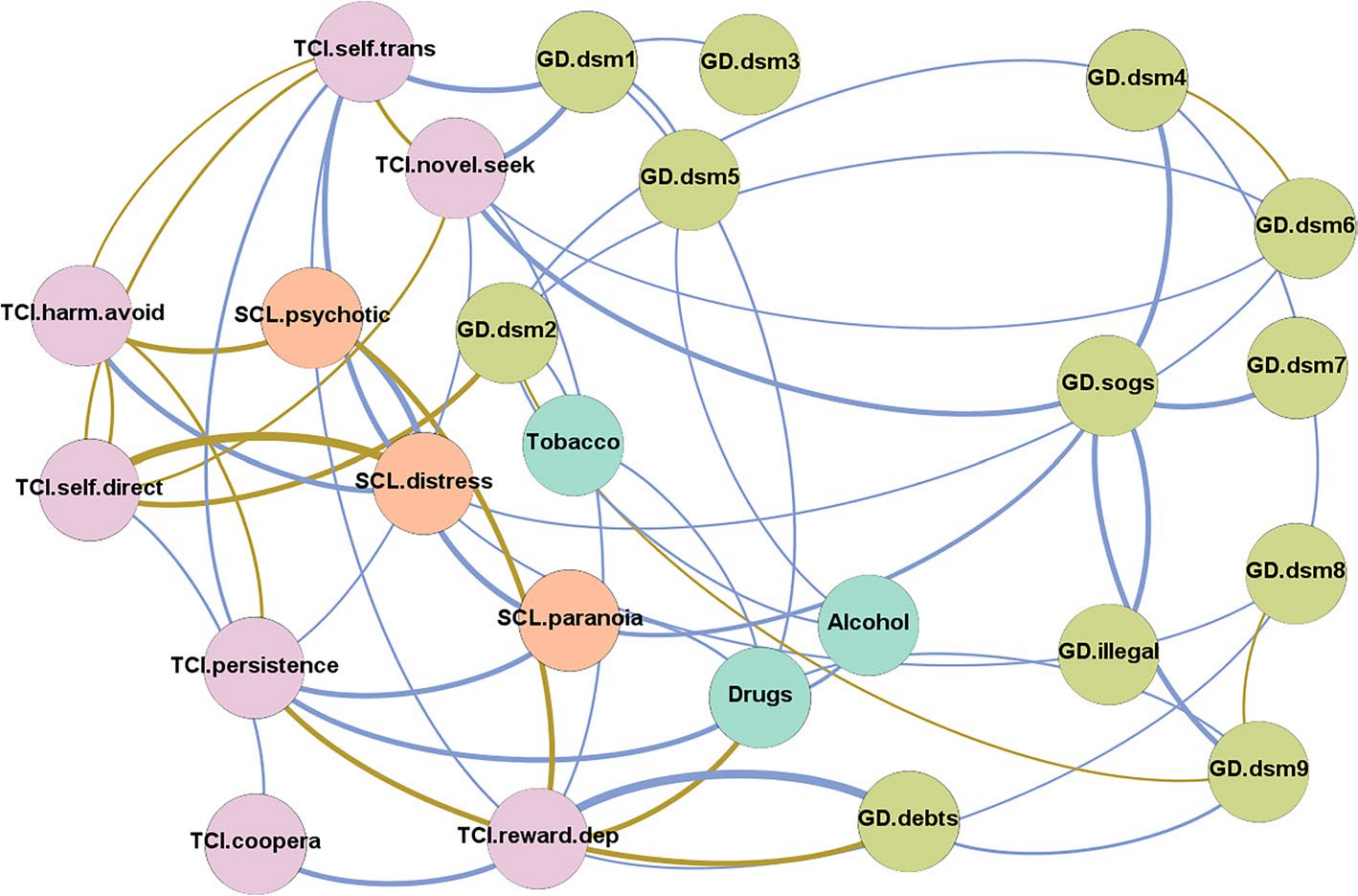
Visualization of the network. *Note*. Positive edges are represented by blue lines, and negative edges are plotted in brown-ochre. As thicker the edge, as stronger the connection weight. Nodes are plotted in colors depending on the dimension: personality (purple), psychopathology (orange), gambling-related measures (light blue), substances (pistachio). Nodes: DSM-5 symptoms for gambling disorder (GD.dsm1 to GD.dsm9), debts related with gambling (GD.debts), illegal behavior related with gambling (GD.illegal), GD symptom level (GD.sogs), global psychopathology distress (SCL.distress), paranoid ideation (SCL.paranoia), psychotic ideation(SCL.psychotic), substances (tobacco, alcohol, and drugs), novelty seeking (TCI_NS), harm avoidance (TCI_HA), reward dependence (TCI_RD), persistence (TCI_PE), self-directedness (TCI_SD), cooperativeness (TCI_CO), self-transcendence (TCI_ST), Tobacco (tobacco use), Alcohol (alcohol use), Drugs (drug use)

The graph of the network obtained in the study is shown in Fig. 1 (the statistics for this analysis are included in Table S2, supplementary material). The diameter achieved a value equal to 4 and the average path length was 2.297.

**Fig. 2:**
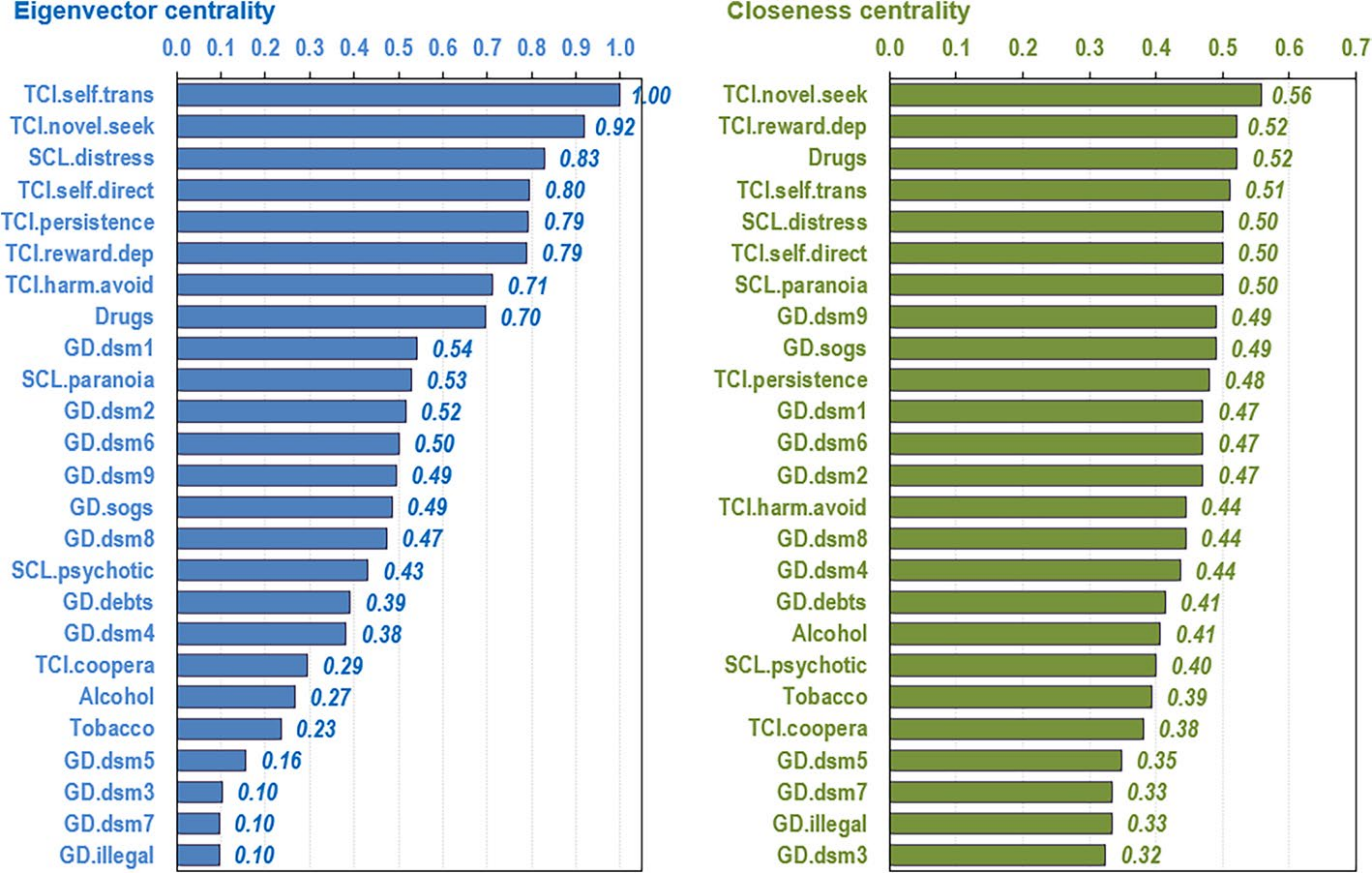
Relevance of centrality and linkage of the nodes. *Note*. Nodes: DSM-5 symptoms for gambling disorder (GD.dsm1 to GD.dsm9), debts related with gambling (GD.debts), illegal behavior related with gambling (GD.illegal), GD symptom level (GD.sogs), global psychopathology distress (SCL.distress), paranoid ideation (SCL.paranoia), psychotic ideation(SCL.psychotic), substances (tobacco, alcohol, and drugs), novelty seeking (TCI_NS), harm avoidance (TCI_HA), reward dependence (TCI_RD), persistence (TCI_PE), self-directedness (TCI_SD), cooperativeness (TCI_CO), self-transcendence (TCI_ST), Tobacco (tobacco use), Alcohol (alcohol use), Drugs (drug use)

The first bar chart in Fig. 2 shows the nodes ordered by eigenvector centrality, which provides a measure of the relevance of each variable in the network. Personality profile, global psychology distress, and the use of illegal drugs achieved the greatest influence. Concretely, self-transcendence and novelty seeking were the nodes with the greatest influence.

The second bar chart in Fig. 2 shows the nodes ordered by closeness centrality, which constitutes a measure of linkage capacity, calculated as the reciprocal of the sum of the length of the shortest paths between the node and all other nodes in the graphon. In this analysis, personality traits and use of drugs also achieved the highest linkage. Concretely, novelty seeking was the closest node to all others in the network.

**Fig. 3:**
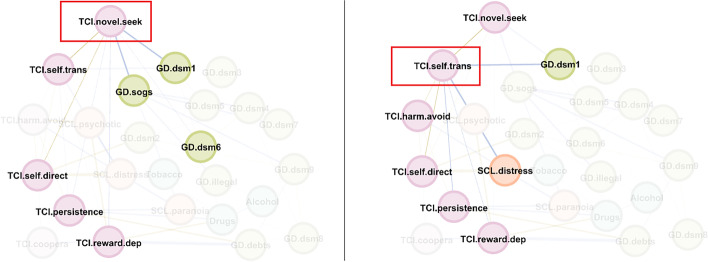
Main linkages for the variables with the highest closeness centrality and eigenvector centrality. *Note*. Nodes: DSM-5 symptoms for gambling disorder (GD.dsm1 to GD.dsm9), debts related with gambling (GD.debts), illegal behavior related with gambling (GD.illegal), GD symptom level (GD.sogs), global psychopathology distress (SCL.distress), paranoid ideation (SCL.paranoia), psychotic ideation(SCL.psychotic), substances (tobacco, alcohol, and drugs), novelty seeking (TCI_NS), harm avoidance (TCI_HA), reward dependence (TCI_RD), persistence (TCI_PE), self-directedness (TCI_SD), cooperativeness (TCI_CO), self-transcendence (TCI_ST), Tobacco (tobacco use), Alcohol (alcohol use), Drugs (drug use)

Figure 3 shows the main linkage for the two variables of greatest centrality in the study (eigenvector and closeness). The activation of the novelty seeking node had a major impact on the other personality traits (except for cooperativeness), the DSM-5 criteria for GD 1 “gambling with increasing amounts of money,” and 6 “chasing one’s losses”, and the GD symptom severity level (SOGS total score). Activation of the “self-transcendence” node also had a major impact on the remaining personality traits (except for cooperativeness), as well as DSM-5 criterion 1 for GD (“gambling with increasing amounts of money”) and global psychology distress (SCL-90R GSI).

**Fig. 4:**
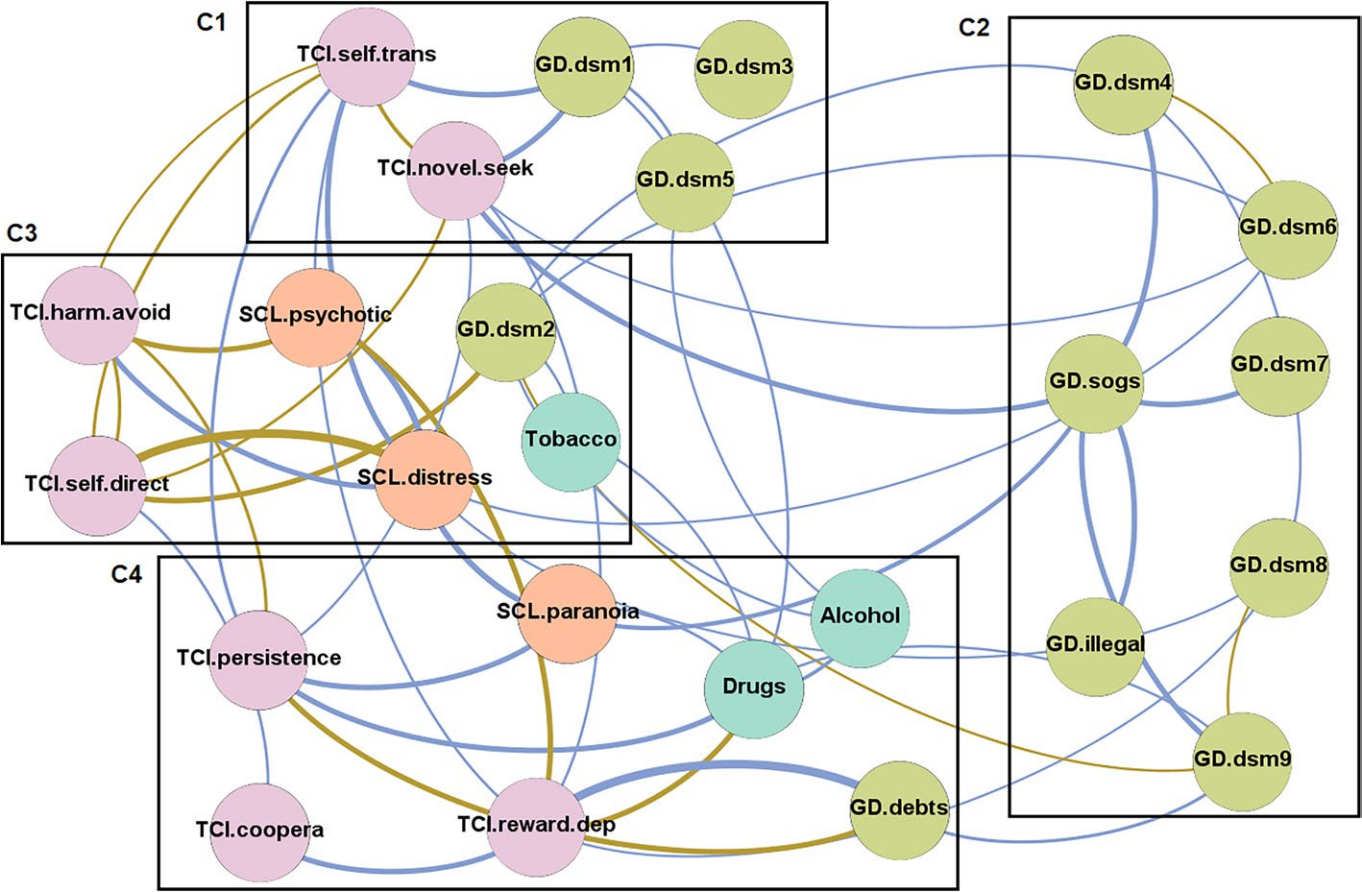
Network grouping the nodes within module-class-clusters. *Note*. Positive edges are represented by blue lines, and negative edges are plotted in brown-ochre. As thicker the edge, as stronger the connection weight. Nodes are plotted in colors depending on the dimension: personality (purple), psychopathology (orange), gambling-related measures (light blue), substances (pistachio). Nodes: DSM-5 symptoms for gambling disorder (GD.dsm1 to GD.dsm9), debts related with gambling (GD.debts), illegal behavior related with gambling (GD.illegal), GD symptom level (GD.sogs), global psychopathology distress (SCL.distress), paranoid ideation (SCL.paranoia), psychotic ideation(SCL.psychotic) , substances (tobacco, alcohol, and drugs), novelty seeking (TCI_NS), harm avoidance (TCI_HA), reward dependence (TCI_RD), persistence (TCI_PE), self-directedness (TCI_SD), cooperativeness (TCI_CO), self-transcendence (TCI_ST), Tobacco (tobacco use), Alcohol (alcohol use), Drugs (drug use)

Four latent modularities (clusters of nodes) were identified (Fig. 4 shows the nodes grouped into each cluster). Cluster 1 (C1) included self-transcendence, novelty seeking, and the DSM-5 criteria for GD 1 “gambling with increasing amounts of money,” 3 “repeated efforts to control-stop gambling,” and 5 “gambling when feeling distressed”. Cluster 2 (C2) grouped another five DSM-5 criteria for GD (criterion 4 “gambling-related preoccupations,” 6 “chasing one’s losses,” 7 “lies related to the gambling impacts,” 8 “losses of relationships, job, or educational opportunities,” and 9 “lies related with financial problems”), the GD symptom level (total SOGS), and gambling-related illegal behaviors. Cluster 3 (C3) grouped the nodes harm avoidance, self-directedness, the DSM-5 criterion for GD 2 “restless-irritable when stopping gambling,” psychotic ideation level, global distress, and tobacco use. Cluster 4 (C4) included the nodes persistence, cooperativeness, reward dependence, paranoid ideation level, gambling-related debts, and the use of drugs and alcohol.

### Network Model Among the Men Sub-sample

Figure S1 (supplementary material) shows the graph of the network obtained among the men sub-sample (*n=163*), and the bar chart with the nodes ordered by the eigenvector centrality and the closeness centrality. The diameter achieved a value equal to 4 and the average path length was 2.397. The node with the highest relevance was self-transcendence, followed by novelty seeking. The node with the highest linkage capacity was novelty seeking, followed by the drug use. Regarding the latent modularities, 5 latent clusters of nodes emerged: C1 included self-transcendence, novelty seeking, persistence, and the DSM-5 criteria for GD 1 “gambling with increasing amounts of money,” 3 “repeated efforts to control-stop gambling,” and 5 “gambling when feeling distressed”; C2 grouped four DSM-5 criteria for GD (criterion 4 “gambling-related preoccupations,” 7 “lies related to the gambling impacts,” 8 “losses of relationships, job, or educational opportunities,” and 9 “lies related with financial problems”), the GD symptom level (total SOGS), and gambling-related illegal behaviors and debts; C3 included harm avoidance, self-directedness, and the SCL-90R scales measuring paranoia ideation, psychotic ideation, and global distress; C4 grouped criterion for GD 2 “restless-irritable when stopping gambling” with the substances; and C5 was defined for cooperativeness and reward dependence.

## Discussion

This study has explored the network structure of the core symptoms for GD, gambling-related impairment measures (debts and illegal acts), psychotic and paranoid ideation levels, emotional distress, substance use, and personality profile, among treatment-seeking patients with comorbid GD plus SCZ. Among the total sample, the variables with the highest impact on the underlying structure were two personality traits: novelty seeking and self-transcendence. Four modularity classes were identified, each including nodes with information on different functionality domains. Among the men sub-sample, the highest relevance was obtained for the nodes self-transcendence and novelty seeking, and the highest linkage capacity was achieved for novelty seeking followed by the use of drugs.

Two personality characteristics appeared as the nodes with the highest centrality indexes, self-transcendence and novelty seeking. Several studies have identified the major contribution of different personality domains to the onset and progression of multiple mental disorders, including schizophrenia and gambling-related problems. Research investigating the influence of personality domains on these two mental disorders has related patients’ risk decisions/choices to the big five factor personality theory (Buelow & Cayton, 2020). Concretely, high neuroticism and low conscientiousness seem to be related to risk-adverse selections in patients with problematic gambling (Brunborg et al., 2016; Takeuchi et al., 2016; Teal et al., 2021). Poor schizophrenia outcomes in terms of symptomatology and quality of life have also been linked with high neuroticism and low extraversion levels (Franquillo et al., 2021), and these two personality features have also helped to explain the heterogeneity within the psychotic disorder spectrum and to conceptualize their comorbidities (Cicero et al., 2019). Therefore, identification of the personality traits implied in patients’ clinical profiles, and the directions and strengths of the associations, is crucial in order to design precise treatment plans and optimize the effectiveness of psychiatric interventions.

In this study, self-transcendence was a central node of the network. As defined in the TCI-R questionnaire, this measure of the individuals’ character covers several factors such as self-forgetfulness, unconscientiousness, and dissolution of the self in experience (Schimmenti et al., 2017). When compared with healthy control samples, patients with high scores for self-transcendence exhibit a profile characterized by negative emotions, introversion (closeness to experience), and absorption (losing track of time and space, focusing all attention on a specific task, and disregarding any other external or internal stimuli) (Anglim et al., 2020; Cloninger et al., 1993; Rezaei et al., 2020). High scores for self-transcendence have been linked to psychotic tendencies and paranoid-schizotypal traits among population-based and clinical samples, especially the cognitive-perceptual component associated with magical thinking (Galindo et al., 2016) and unusual perceptions (such as delusions and bizarre-unconventional beliefs) (Miskovic et al., 2018). This profile is typical of emotionally unstable individuals with a tendency to perceive any event as potentially stressful, and therefore highly vulnerable to anxiety and to the use of unhealthy behaviors to cope with these negative states (Zhou et al., 2017). And among SCZ patients, self-transcendence has been related with multiple unhealthy correlates, such as the social function (troubled and non-satisfying interpersonal relationships) (Kashiwagi et al., 2022), suicidal behavior (Canal-Rivero et al., 2021), and the presence of dual diagnoses characterized by substance-related disorder (Río-Martínez et al., 2020). These results could elucidate the results obtained in our network, suggesting that self-transcendence among SCZ could increase patients’ propensity to engage in risky situations, while predisposing them in an isolated-closed world (these are, precisely, two relevant signs of the onset and progression of gambling-related problems). In addition, based on the connections between self-transcendence and negative affectivity, detachment and poor social support observed in previous studies, high scores in this personality domain could act as a “bridge node” in SCZ patients towards uncontrolled gambling as a way to inhibit harmful feelings (Kayiş et al., 2016; Laier et al., 2018; Müller et al., 2014; Wittek et al., 2016). It is likelihood that context that accompanies gambling behaviors could be perceived by SCZ patients as a safe environment in which they are not exposed to ridicule (or rejected), since face-to-face social skills are not required. And since the preferred form of gambling among SCZ patients is largely non-strategic face-to-face games, especially slot machines (which do not require special skills or technological knowledge), while they are gambling patients are not exposed to the failure they usually perceive in their real-life experiences.

Novelty seeking also achieved a very high centrality index in the network modeled in this work. Measured by the TCI-R, this personality trait is defined as the tendency towards excitement and exploratory activity in response to novel stimulation, avoidance of frustration, and impulsive decision-making (Cloninger et al., 1993). Previous studies observed that this trait is strongly related to impulsiveness, a complex multifaceted trait proposed as a vulnerability marker (Marín-Navarrete et al., 2018) and a transdiagnostic sign within multiple mental disorders (Dalley & Robbins, 2017), including SCZ (Peritogiannis, 2015; Şenormanci et al., 2022) and GD (Szerman et al., 2020). Impulsiveness has also been proven in previous research to be a key feature for explaining the correlations between SCZ and multiple psychiatric symptoms, such as substance use (Dondé et al., 2020) and GD (Granero et al., 2021; Hodgins & Holub, 2015; Kräplin et al., 2014; Lee et al., 2013; Ouzir, 2013). In this study, novelty seeking obtained the highest closeness centrality, and this suggests that it could be a strong transition-bridge node within the comorbid profile of SCZ and GD patients. In network analysis, nodes with high transition-bridge capacity are interpreted as triggers of symptoms/signs from different disorders, thus promoting psychiatric comorbidity (Fried et al., 2017; Fried & Cramer, 2017). Since this study analyzed a sample of patients with a dual comorbid condition, high transition-bridge capacity could be interpreted as expanding the activation of other nodes related with the worst functional state. Concretely, the activation of novelty seeking in this work strongly impacted the total SOGS score (a measure of GD symptom severity), but also DSM-5 criterion 1 for GD (“gambling with increasing amounts of money,” a tolerance measure) and DSM-5 criterion 6 for GD (“chasing one’s losses,” one of the central characteristics of problematic gambling). Other studies have found that in SCZ or schizoaffective disorder, chasing was associated with greater gambling involvement and with greater problems with substance use (Yakovenko et al., 2018; Yakovenko & Hodgins, 2018). Impulsivity has also been identified as the most relevant personality trait associated to the presence of GD and the severity of this disorder (Ioannidis et al., 2019).

It must be also outlined that cognitive inhibitory control deficits associated with impulsivity have been linked with psychopathological disorders such as addictive behaviors and SCZ (Dixon et al., 2022). Medication use is other related important factor, since impulsive responses have been associated in some patients within the impulsive spectrum disorders with dopamine agonist therapy during chronic treatments (Napier & Persons, 2019). Pharmacological research published in the last 20 years has related the increase in impulsivity-related problems in SCZ patients to dose response to some dopamine agonist drugs (Kim et al., 2021; Kishi et al., 2021; Pahwa et al., 2021), including clinical cases with dual mental conditions (Clerici et al., 2018). These studies show that aripiprazole (an atypical anti-psychotic drug), as well as other dopamine replacement therapies, contributes to the development of impulsive responses, and these medications therefore constitute risk factors for the onset and progression of GD among SCZ populations. This specific association has been explained by the hyperdopaminergic state in the mesolimbic pathway (reward system) through the predominant action on dopamine D2 and D3 receptors of these medications (Tuplin & Holahan, 2017). But while these studies are consistent with the relevance of impulsiveness among patients with the dual condition of SCZ with GD, the results must be considered with caution: although aripiprazole could contribute to the onset of gambling-related problems in the early stages of SCZ, the causality, strength (effect size), and specific process (direct, indirect, or mixed effect) have not been proven in terms of causality (Qian et al., 2021). The impact of long-term medication use on the neurometabolite levels is still not fully understood among SCZ patients, and future longitudinal research is needed to analyze the complex relationships between specific changes due to medication uses from specific disease-related changes. On the other hand, the pharmacological plan (medication and dose) was not available for SCZ in this study, which does not permit attribution of the central role of impulsivity to a potential dose response to antipsychotic treatments.

Regarding the analysis of modules-clusters in this study, it should be noted that the procedure used in the network approach is different from conventional classification analysis. For example, analytical techniques such as k-means or two-step-cluster are focused on the grouping of elements (usually individuals) by the degree of similarity-difference between the set of components. However, the network approach identifies empirical groups of nodes (which can be defined for individuals, but also for variables) based on their edges (the key element is the linkage) and their attributes (identity relationships between classes). The evidence of different modules/clusters in a network suggests that groups of nodes could be part of specific processes, with the condition that some of these nodes may be closely interacting with nodes pertaining to other processes (that is, high linkage is possible intra-cluster and also between clusters). And although some of the modules/clusters may include sets of symptoms/criteria as defined in classical categorical taxonomies such as the DSM or the ICD, the activation of a node belonging to a module can transcend the cluster itself, thus increasing the likelihood of activating related nodes belonging to other groups (Borsboom, 2017; Boschloo et al., 2015). This would be, precisely, the role of “bridge nodes,” which in clinical research are usually called “transdiagnostic symptoms.” In this study, four clusters have been identified, and three of them include variables that measure patients’ heterogeneous domains. Specifically, cluster C1 grouped the two personality variables with the highest centrality indexes in the study (self-transcendence and novelty seeking), along with three DSM-5 symptoms for GD. Clusters C3 and C4 grouped personality factors, DSM-5 criteria for GD, variables related to substance use, and psychopathology levels according to the SCL-90R.

### Limitations and Strengths

This study should be interpreted in the context of some limitations. First, the network has been modeled with several nodes that measured multiple aspects of the psychological and functional areas. However, other nodes that previous research relates to the GD and SCZ phenotypes were not available for this study (such as specific neurological and physical variables), so it was not possible to assess their contribution to the pattern of the relationships. In relation with this first limitation, in our network, the nodes related to the core/specific symptoms of GD were overrepresented compared to the nodes related to the core/specific symptoms of SCZ (the complete list of DSM-5 criteria for SCZ was not available), and this hinders the ability to identify with greater reliability-validity the true central and bridge nodes among the sample of patients with the comorbid condition GD and SCZ (after all, any analytical procedure analysis is be highly influenced by the variables analyzed). Future studies should explore the centrality (relevance and linkage) of alternative networks with additional nodes specifically focused on the SCZ profile.

Second, the sample in this study can be considered low in view of the analytical plan, but large in view of the specific composition (treatment-seeking patients with dual GD and SCZ, a comorbid condition uncommon even in tertiary treatment centers specialized in behavioral addictions). The sex distribution was asymmetrical (the number of women was particularly low), but this distribution is consistent with the ratio of men/women observed in clinical settings (any case, the limited proportion of female participants must be considered for generalization purposes). Regarding the impact of the sample size on the statistical procedures, it must also be outlined that there is no consensus rule on the optimal number of participants to ensure the reliability and validity of the network analysis, but like other statistical analysis, it is important that the sample size allows to accurately estimate the statistical parameters for safeguarding against erroneous conclusions which contribute to the robustness and replicability of the research. A current simulation study has observed that sample requirements for network are into a very broad range depending on different design factors, such as the network architecture, network connectedness, number of nodes, and type of data (Constantin et al., 2022). Our study has been performed with large number of nodes and edges, and this could impact in the capacity of the model to achieve accurate parameters. Therefore, our results must be interpreted with caution, in the context of being a pioneering study whose results must be corroborated/refuted by future studies with larger samples.

Third, the cross-sectional nature of the data does not enable interpretation of the results in causal terms (in this study, the network was modeled by defining undirected edges). Finally, the lack of previous research on the subject matter (network studies applied to the etiology of GD and SCZ) impacts the interpretation of the findings, since there is no solid theoretical framework with which to contextualize the new empirical evidence generated by this study.

The last limitation is the long recruitment period for the sample. This is a typical procedure in studies analyzing (un)healthy conditions with few prevalence even in specialized clinical settings. In our study, the selection of all the participants was done according to the same inclusion-exclusion criteria, the same assessment tools were used, and the diagnostic of GD was homogeneous (based on the DSM-5 criteria). However, sociocultural changes have occurred during the recruitment period (including the COVID-19 pandemic), as well as pharmacological/treatment plans for SCZ. Related with this fact, the specific antipsychotic medication was not registered and controlled (nor the presence/impact of the negative symptoms). Future research should examine the potential interactive contribution of these measures, which should be considered in the interpretation of the results of our study.

This study has various strengths. First, there is the use of the network methodology to visualize the structure of interrelations between nodes and modules-clusters, and to identify the central nodes/variables in the clinical profile of patients with the comorbid/dual profile of GD and SCZ (this is a clinical condition that has a considerable effect on the patients’ functionality level). One advantage of network analysis is its focus on the concrete nodes/variables that form the complete clinical profile (instead of focusing on single underlying latent constructs, the disorders), with the aim of measuring the relevance and closeness of each node, providing the pattern of relationships and exploring potential sub-groups.

## Conclusions

To the best of our knowledge, this is the first study to focus on the analysis of centrality (relevance and linkage) within the profile of patients with SCZ who are receiving treatment for gambling-related problems. The role of personality traits as the most central features, the identification of distinct modularity classes (grouping nodes that contain information from different functional dimensions), and the patterns of interrelations between the nodes support the hypothesis of a complex process sustaining the clinical profile. These results illustrate the difficulty of conceptualizing GD and SCZ as two well-defined, separate nosological entities, acting as single latent factors. On the contrary, the clinical phenotype of patients with GD and SCZ seems to be the result of a dense pattern of associations between psychological symptoms, personality traits, and other functional measures. And this complex structure suggests the involvement of common neurobiological impairments and the affectation of common brain systems, which should be participating in the expression of the distinct mental disorders. Therefore, profiles such as dual conditions should be approached from a transdiagnostic perspective, including personality traits such as impulsivity.

Finally, the objective of this study was not to assess the structure of the core symptoms/criteria for GD and SCZ as described in categorical taxonomies such as ICD-11 or DSM-5, but to explore the network of these core symptoms plus other sets of variables measuring the gambling-related impacts, the concurrence of substance uses, and personality traits. The conclusions of the study must also be contextualized to the specific population of individuals with the comorbid condition of SCZ with GD. Among these patients, the assessment of the contribution of the multiple nodes (relevance, linkage capacity, and clustering in modularity classes) provides evidence regarding the endophenotypes of GD and SCZ and suggests the suitability of a paradigm shift in the way that patients with this dual condition are conceptualized, diagnosed, and treated. Upcoming studies should also validate the underlying structure of symptoms in samples of pure SCZ and pure GD patients. Future research is crucial, particularly studies focused on clinical neuroscience and precision psychiatry.

## Supplementary Information

Figure S1 Visualization of the network among the men sub-sample. Note. Positive edges are represented by blue lines, and negative edges are plotted in brown-ochre. As thicker the edge as stronger the connection weight. Nodes are plotted in colors depending on the dimension. Nodes: DSM-5 symptoms for gambling disorder (GD.dsm1 to GD.dsm9), debts related with gambling (GD.debts), illegal behavior related with gambling (GD.illegal), GD symptom level (GD.sogs), global psychopathology distress (SCL.distress), paranoid ideation (SCL.paranoia), psychotic ideation(SCL.psychotic) , substances (Tobacco, Alcohol and Drugs), novelty seeking (TCI_NS), harm avoidance (TCI_HA), reward dependence (TCI_RD), persistence (TCI_PE), self-directedness (TCI_SD), cooperativeness (TCI_CO), self-transcendence (TCI_ST), Tobacco (tobacco use), Alcohol (Alcohol use), Drugs (drugs use). (PNG 2563 kb)

High Resolution Image (TIF 615 kb)

Supplementary file 1 (DOCX 460 kb)
