# Supplementary figures and images for: Network Analysis of the Structure of the Core Symptoms and Clinical Correlates in Comorbid Schizophrenia and Gambling Disorder
Source: Int J Ment Health Addict. 2022 Dec 27:1–27. Online ahead of print. doi: 10.1007/s11469-022-00983-y (PMC9794112; doi:10.1007/s11469-022-00983-y)

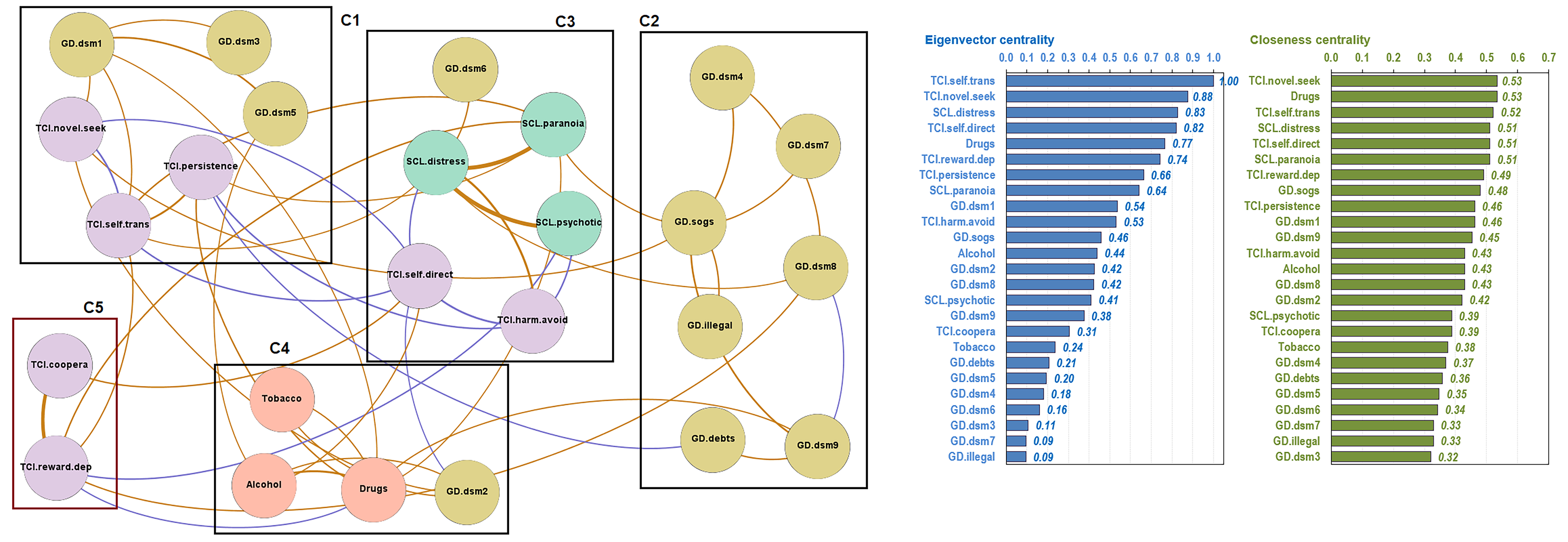

Supplement: Supplementary file 1 — Visualization of the network among the men sub-sample. Note. Positive edges are represented by blue lines, and negative edges are plotted in brown-ochre. As thicker the edge as stronger the connection weight. Nodes are plotted in colors depending on the dimension. Nodes: DSM-5 symptoms for gambling disorder (GD.dsm1 to GD.dsm9), debts related with gambling (GD.debts), illegal behavior related with gambling (GD.illegal), GD symptom level (GD.sogs), global psychopathology distress (SCL.distress), paranoid ideation (SCL.paranoia), psychotic ideation(SCL.psychotic) , substances (Tobacco, Alcohol and Drugs), novelty seeking (TCI_NS), harm avoidance (TCI_HA), reward dependence (TCI_RD), persistence (TCI_PE), self-directedness (TCI_SD), cooperativeness (TCI_CO), self-transcendence (TCI_ST), Tobacco (tobacco use), Alcohol (Alcohol use), Drugs (drugs use). (PNG 2563 kb) [file 11469_2022_983_Fig5_ESM.png]

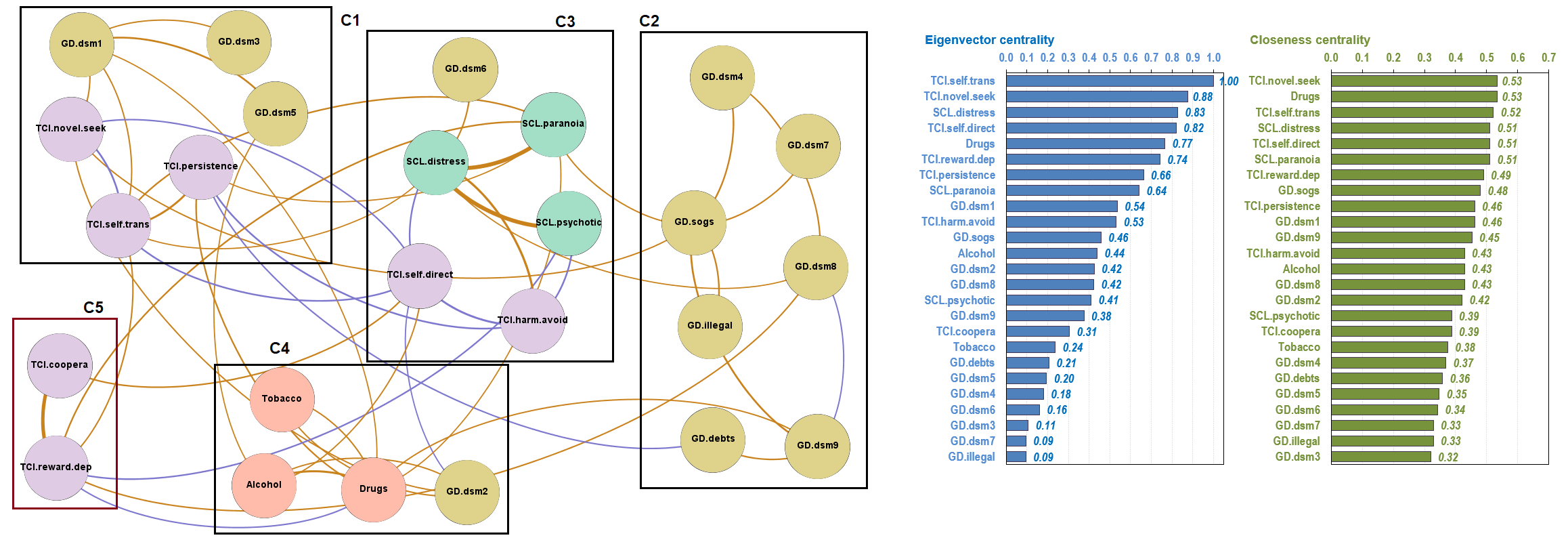

Supplement: Supplementary file 2 — High Resolution Image (TIF 615 kb) [file 11469_2022_983_MOESM1_ESM.tif]
