# Supplementary material for: Network Analysis of the Structure of the Core Symptoms and Clinical Correlates in Comorbid Schizophrenia and Gambling Disorder
Source: Int J Ment Health Addict. 2022 Dec 27:1–27. Online ahead of print. doi: 10.1007/s11469-022-00983-y (PMC9794112; doi:10.1007/s11469-022-00983-y)
Supplement: Supplementary file 3 — (DOCX 460 kb) [file 11469_2022_983_MOESM2_ESM.docx]

*Table S1 Comparison between sexes for the variables of the study*

|  | Women *(n=16)* | | Men *(n=163)* | |  |
| --- | --- | --- | --- | --- | --- |
| *Sociodemographic* | *n* | *%* | *n* | *%* | *p* |
| Marital Single | 10 | 62.5% | 113 | 69.3% | .706 |
| Married | 3 | 18.8% | 31 | 19.0% |  |
| Divorced | 3 | 18.8% | 19 | 11.7% |  |
| Education Primary | 7 | 43.8% | 109 | 66.9% | .157 |
| Secondary | 9 | 56.3% | 49 | 30.1% |  |
| University | 0 | 0.0% | 5 | 3.1% |  |
| Employed Unemployed | 13 | 81.3% | 127 | 77.9% | .758 |
| Employed | 3 | 18.8% | 36 | 22.1% |  |
| Social position Mean-high | 0 | 0.0% | 3 | 1.8% | .575 |
| Mean | 0 | 0.0% | 7 | 4.3% |  |
| Mean-low | 7 | 43.8% | 33 | 20.2% |  |
| Low | 9 | 56.3% | 120 | 73.6% |  |
| Born in… Spain | 15 | 93.8% | 153 | 93.9% | .985 |
| Other | 1 | 6.3% | 10 | 6.1% |  |
| *Age and duration of GD* | *Mean* | *SD* | *Mean* | *SD* | *p* |
| Age (yrs-old) | 41.94 | 8.18 | 39.29 | 10.08 | .311 |
| Duration of GD (yrs-old) | 5.75 | 5.72 | 6.96 | 6.97 | .501 |
| *Psychopathology (SCL-90R)* | *Mean* | *SD* | *Mean* | *SD* | *p* |
| Paranoid ideation | 1.31 | 0.72 | 1.30 | 0.86 | .959 |
| Psychotic ideation | 1.30 | 0.78 | 1.21 | 0.91 | .710 |
| Global distress (GSI) | 1.43 | 0.62 | 1.33 | 0.76 | .608 |
| *Personality (TCI-R)* | *Mean* | *SD* | *Mean* | *SD* | *p* |
| Novelty seeking | 107.06 | 15.62 | 108.93 | 9.55 | .485 |
| Harm avoidance | 111.25 | 18.59 | 108.67 | 12.94 | .467 |
| Reward dependence | 94.31 | 7.35 | 94.12 | 11.77 | .948 |
| Persistence | 103.50 | 16.78 | 103.06 | 17.86 | .924 |
| Self-directedness | 116.06 | 14.29 | 121.48 | 17.62 | .235 |
| Cooperativeness | 127.38 | 11.79 | 126.06 | 14.30 | .723 |
| Self-transcendence | 72.06 | 15.75 | 64.28 | 13.45 | .061 |
| *GD measures* | *n* | *%* | *n* | *%* | *p* |
| A1: Gambling with increasing amounts of money | 13 | 81.3% | 121 | 74.2% | .537 |
| A2: Restless-irritable when stop gambling | 15 | 93.8% | 143 | 87.7% | .475 |
| A3: Repeated efforts to control-stop gambling | 16 | 100.0% | 150 | 92.0% | .241 |
| A4: Preoccupied with gambling | 13 | 81.3% | 122 | 74.8% | .570 |
| A5: Often gambles when feeling distressed | 14 | 87.5% | 114 | 69.9% | .138 |
| A6: Chasing one’s losses | 13 | 81.3% | 129 | 79.1% | .842 |
| A7: Lies to conceal the extent of gambling | 14 | 87.5% | 147 | 90.2% | .733 |
| A8: Has lost relationships, job, education | 14 | 87.5% | 146 | 89.6% | .797 |
| A9: Relies related with financial issues | 12 | 75.0% | 138 | 84.7% | .317 |
| Preference Only non-strategic forms of gambling | 14 | 87.5% | 122 | 74.8% | .394 |
| Only strategic forms of gambling | 1 | 6.3% | 8 | 4.9% |  |
| Mixed forms of gambling | 1 | 6.3% | 33 | 20.2% |  |
| Modality Only land-based | 15 | 93.8% | 150 | 92.0% | .626 |
| Only online | 0 | 0.0% | 7 | 4.3% |  |
| Mixed | 1 | 6.3% | 6 | 3.7% |  |
| Debts due to GD (yes) | 7 | 43.8% | 73 | 44.8% | .937 |
| Illegal behavior (yes) | 2 | 12.5% | 51 | 31.3% | .116 |
|  | *Mean* | *SD* | *Mean* | *SD* | *p* |
| SOGS total score | 10.34 | 2.50 | 10.77 | 2.77 | .586 |
| *Substances* | *n* | *%* | *n* | *%* | *p* |
| Tobacco | 13 | 81.3% | 127 | 77.9% | .758 |
| Alcohol | 2 | 12.5% | 29 | 17.8% | .594 |
| Illegal drugs | 1 | 6.3% | 25 | 15.3% | .325 |

*Table S1 (Supplementary)*

*Results of the network*

| ID | Dimension | Label | Eigenvector.  centrality | Authority | Closeness.  centrality | Harm.Clos. Centrality | Betweeness.  centrality | Modularity.  Class | Clustering.  Coefficient | HUB | Number.  Triangles |
| --- | --- | --- | --- | --- | --- | --- | --- | --- | --- | --- | --- |
| GD.dsm1 | Gambling | DSM-5 criterion 1 | 0.541860 | 0.191391 | 0.470588 | 0.548611 | 39.343506 | 1 | 0.100000 | 0.191385 | 1 |
| GD.dsm2 | Gambling | DSM-5 criterion 2 | 0.515836 | 0.172312 | 0.470588 | 0.565972 | 26.833766 | 3 | 0.066667 | 0.172316 | 1 |
| GD.dsm3 | Gambling | DSM-5 criterion 3 | 0.102877 | 0.035961 | 0.324324 | 0.361111 | 0.000000 | 1 | 0.000000 | 0.035962 | 0 |
| GD.dsm4 | Gambling | DSM-5 criterion 4 | 0.382551 | 0.126395 | 0.436364 | 0.510417 | 7.853968 | 2 | 0.166667 | 0.126392 | 1 |
| GD.dsm5 | Gambling | DSM-5 criterion 5 | 0.156877 | 0.052324 | 0.347826 | 0.402778 | 1.652381 | 1 | 0.000000 | 0.052327 | 0 |
| GD.dsm6 | Gambling | DSM-5 criterion 6 | 0.501299 | 0.174051 | 0.470588 | 0.534722 | 6.686905 | 2 | 0.166667 | 0.174048 | 1 |
| GD.dsm7 | Gambling | DSM-5 criterion 7 | 0.095428 | 0.030538 | 0.333333 | 0.371528 | 0.000000 | 2 | 0.000000 | 0.030538 | 0 |
| GD.dsm8 | Gambling | DSM-5 criterion 8 | 0.473891 | 0.163770 | 0.444444 | 0.520833 | 7.826984 | 2 | 0.000000 | 0.163775 | 0 |
| GD.dsm9 | Gambling | DSM-5 criterion 9 | 0.494924 | 0.164350 | 0.489796 | 0.562500 | 20.325902 | 2 | 0.000000 | 0.164343 | 0 |
| GD.sogs | Gambling | SOGS total score | 0.485004 | 0.162523 | 0.489796 | 0.576389 | 51.599206 | 2 | 0.000000 | 0.162521 | 0 |
| GD.debts | Gambling | Debts due to gambling | 0.391654 | 0.137190 | 0.413793 | 0.479167 | 2.645635 | 4 | 0.000000 | 0.137196 | 0 |
| GD.illegal | Gambling | Illegal behavior | 0.095428 | 0.030538 | 0.333333 | 0.371528 | 0.000000 | 2 | 0.000000 | 0.030538 | 0 |
| Tobacco | Substances | Tobacco use/abuse | 0.234478 | 0.077258 | 0.393443 | 0.444444 | 1.355952 | 3 | 0.000000 | 0.077254 | 0 |
| Alcohol | Substances | Alcohol use/abuse | 0.266959 | 0.087090 | 0.406780 | 0.472222 | 8.568795 | 4 | 0.000000 | 0.087086 | 0 |
| Drugs | Substances | Illegal drugs use/abuse | 0.695457 | 0.238830 | 0.521739 | 0.611111 | 42.341883 | 4 | 0.047619 | 0.238838 | 1 |
| SCL.paranoia | Psychopathology | SCL-90R Paranoia | 0.529351 | 0.185195 | 0.500000 | 0.555556 | 13.156349 | 4 | 0.166667 | 0.185196 | 1 |
| SCL.psychotic | Psychopathology | SCL-90R Psychotic | 0.431925 | 0.158359 | 0.400000 | 0.472222 | 0.976190 | 3 | 0.333333 | 0.158362 | 1 |
| SCL.distress | Psychopathology | SCL-90R GSI | 0.828136 | 0.299655 | 0.500000 | 0.597222 | 19.884127 | 3 | 0.190476 | 0.299653 | 4 |
| TCI.novel.seek | Personality | TCI-R Novelty seeking | 0.919356 | 0.327917 | 0.558140 | 0.631944 | 46.293723 | 1 | 0.190476 | 0.327923 | 4 |
| TCI.harm.avoid | Personality | TCI-R Harm avoidance | 0.711902 | 0.261938 | 0.444444 | 0.534722 | 4.148016 | 3 | 0.500000 | 0.261941 | 5 |
| TCI.reward.dep | Personality | TCI-R Reward dependence | 0.789018 | 0.281205 | 0.521739 | 0.611111 | 34.228716 | 4 | 0.047619 | 0.281195 | 1 |
| TCI.persistence | Personality | TCI-R Persistence | 0.793246 | 0.284598 | 0.480000 | 0.569444 | 13.876335 | 4 | 0.200000 | 0.284591 | 3 |
| TCI.self.direct | Personality | TCI-R Self-directedness | 0.795619 | 0.287918 | 0.500000 | 0.583333 | 19.824206 | 3 | 0.266667 | 0.287914 | 4 |
| TCI.coopera | Personality | TCI-R Cooperativeness | 0.294996 | 0.106935 | 0.380952 | 0.437500 | 0.659524 | 4 | 0.000000 | 0.106939 | 0 |
| TCI.self.trans | Personality | TCI-R Self-transcendence | 1.000000 | 0.363510 | 0.510638 | 0.604167 | 18.917929 | 1 | 0.380952 | 0.363517 | 8 |
